# Psychometric properties of Lehman’s quality of life interview and its modifications: a systematic review using the COSMIN methodology

**DOI:** 10.1038/s41537-026-00777-4

**Published:** 2026-06-23

**Authors:** Max Wiessner, Milena Zúñiga Le-Bert, Sophia Wehr, Lucia Weigel, Stefan Leucht

**Affiliations:** 1 Technical University of Munich, TUM School of Medicine and Health, Department of Psychiatry and Psychotherapy, Klinikum rechts der Isar, Munich, Germany; 2 German Center for Mental Health (DZPG), Munich, Germany

**Keywords:** Schizophrenia, Psychiatric disorders

## Abstract

Background: Schizophrenia is a severe mental disorder with significant impact on quality of life (QoL). Reliable measurement of QoL is essential for evaluating treatment outcomes. Methods: We applied the COSMIN (COnsensus-based Standards for the selection of health Measurement INstruments) systematic review guideline to evaluate the psychometric properties of Lehman’s Quality of Life Interview (QOLI), the Lancashire Quality of Life Profile (LQOLP), and the Manchester Short Assessment of Quality of Life (MANSA) as patient-reported outcome measures (PROMs). Results: The combined search of PubMed and EMBASE yielded 19 included studies: 4 for QOLI, 10 for LQOLP, and 4 for MANSA. The overall results of the measurement properties across all three scales are largely insufficient or indeterminate. Evidence for several measurement properties is missing for the individual scales, with studies on content validity in particular being absent for all three scales. Conclusion: All three scales fall in COSMIN category B, meaning they have potential to be recommended but certain important evidence for recommendation is missing. However, newer scales - such as the Schizophrenia Quality of Life Scale - have been developed using more modern approaches. Consequently, prioritizing the further evaluation of these newer instruments may prove more productive than continued research into older scales.

## Introduction

Schizophrenia is a severe mental illness with profound effects on patients’ daily functioning and well-being. Beyond symptom reduction, Quality of Life (QoL) has become an important aspect in treatment and care^1^. Reliable measurement of QoL is therefore essential.

With the aim to provide an instrument to assess the QoL of the chronically mentally ill, Lehman developed the Lehman’s Quality of Life Interview (QOLI)^2^. Further development of Lehman’s original work and adaptation to UK conditions resulted in the Lancashire Quality of Life Profile (LQOLP)^3^. Out of the desire to have a shorter and more compact instrument, the Manchester Short Assessment of Quality of Life (MANSA)^4^ was created as a brief and modified version of the LQOLP.

The QOLI is a structured interview tool designed to assess the QoL in individuals with severe mental illness. It contains 143 items that cover 8 domains (living situation, family relations, social relations, leisure, work, finances, safety, and health). Each domain incorporates both objective and subjective components, allowing for a comprehensive assessment of the individual’s quality of life. The estimated completion time is approximately 45 min^2^.

The LQOLP comprises 105 items covering 9 domains (living situation, family, social relationships, leisure activities, work/education, finances, personal safety, health, and religion). Each domain is scored to reflect both objective conditions and subjective satisfaction, providing a detailed picture of the individual’s quality of life. The mean time of completion is about 33 minutes for experienced clinicians^3^.

MANSA contains 16 questions, four of them investigating objective quality of life and 12 satisfaction with life as a whole, job, financial situation, friendships, leisure activities, accommodation, personal safety, people that the person lives with, family and health. The time of completion is between 3 and 5 min^4^.

To the best of our knowledge, a systematic review of the psychometric properties of the instruments described has not yet been conducted. In view of the above, a systematic review was conducted in accordance with the COnsensus-based Standards for the selection of health Measurement INstruments (COSMIN) guidelines^5–7^.

## Methods

The COSMIN (COnsensus-based Standards for the selection of health Measurement INstruments) methodology is a systematic approach to assess the quality of patient-reported outcome measures (PROMs). By performing this assessment, strengths and weaknesses of outcome measurement instruments can be detected, and evidence-based recommendations can be formulated for clinicians and researchers to decide which instrument is best for a given purpose.

**Fig. 1:**
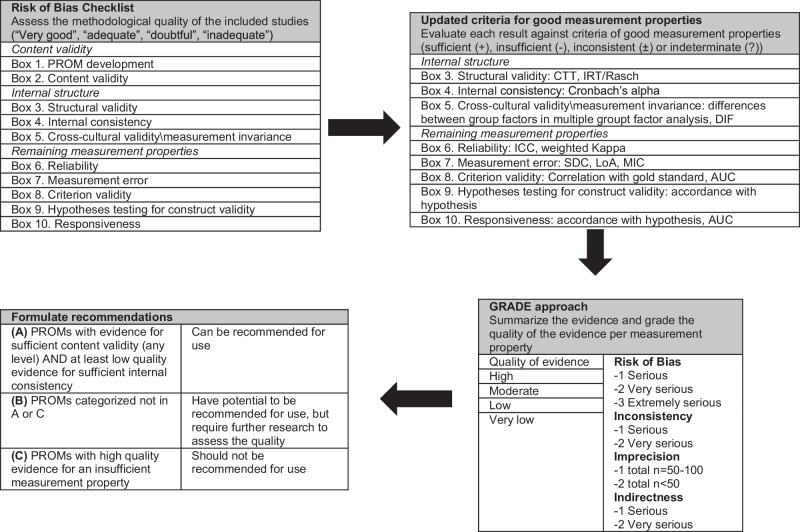
Overview of the COSMIN methodology^5–7^. For further information refer to the methods part of the publication or the COSMIN manual. Step 1: Risk of Bias Checklist. Step 2: Updated criteria for good measurement properties. Step 3: GRADE approach (for further information see Table 1a+b). Step 4: Formulate recommendations.

In short, the methodology consists of literature search, evaluating the particular measurement properties (via Risk of Bias checklist, updated criteria, GRADE approach), and formulating recommendations. An overview of the methodology is given in Fig. 1. Further details on the methodology can be found in the COSMIN checklist and manual^5–7^.

### Protocol registration

The study protocol has been registered with OSF: osf.io/r5bx9.

### Scale selection

Scale selection for our systematic review was guided by van Krugten et al. ^8^, who assessed the extent to which existing QOL instruments cover the domains defined by Connell et al. ^9,10^. These domains encompass key aspects of quality of life important to individuals with mental health conditions. Beyond QOLI, LQOLP, and MANSA, we included scales covering at least a subset of these domains: the Heinrichs-Carpenter QoL Scale, SQoL-41/-18, Wisconsin Quality of Life Index, Quality of Life in Schizophrenia questionnaire, and Schizophrenia Quality of Life Scale (Revision 4). The measurement properties of these scales are reported in separate publications, some of which are already published^11^ and others currently in preparation.

### Literature search

A comprehensive search was conducted in the databases PubMed and Embase. Included were articles published in English up to November 2022. The reference lists of the selected articles were screened as well. The literature search was conducted by two reviewers (MZ, MW) independently. Disparities were discussed until consensus was reached, in doubt consulting third reviewers (LW, SW). The search terms used were ‘Lehmans Quality of Life interview’, ’Quality of Life Interview’, ‘QOLI’ and ‘QLI’ in “Title/Abstract” for QOLI, ‘Lancashire Quality of Life Profile’, ’Quality of Life Profile’, ‘LQOLP’ and ‘QOLP’ in “Title/Abstract” for LQoLP and ‘Manchester Short Assessment of Quality of Life’, ’Manchester Short Assessment’ and ‘MANSA’ in “Title/Abstract” for MANSA. To ensure currency of the database we conducted a supplementary search on PubMed for journal articles in English published between November 2022 and April 2026 in accordance with the COSMIN manual.

### Assessment of the measurement properties

For all three steps the evaluation of the measurement properties was completed by two reviewers (MZ and MW) independently. Dissent was discussed until consensus was reached, in doubt by consulting third reviewers of expert-level (SW, LW).

### Evaluating the risk of bias

To report on the methodological quality of each validation study of an instrument COSMIN developed the Risk of Bias checklist. The checklist contains 10 different boxes with specific criteria to rate the quality of each study on a measurement property separately. The 10 boxes are: PROM development, content validity, structural validity, internal consistency, cross-cultural validity/measurement invariance, reliability, measurement error, criterion validity, hypotheses testing for construct validity and responsiveness.

The first two boxes “PROM development” and “content validity” assess the content validity of an instrument and were found to be relevant solely for the original papers in which the development of an instrument is described.

The evaluation of risk of bias involves assigning scores to each measurement property based on a four-point scale that includes the descriptors “very good,” “adequate,” “doubtful,” and “inadequate.” Additionally, a “not applicable” option was provided for each property. The assessment of relevant measurement properties for each study was conducted, and an overall score for the methodological quality of each property was determined using the Cosmin Risk of Bias Checklist. This checklist employs the “worst score counts” principle^5–7^.

#### Evaluating the updated criteria for good measurement properties

In the second step, the results of the validation studies are evaluated using the ‘updated criteria for good measurement properties’^5–7^. Therefore, results on the measurement properties are extracted. For each measurement property an overall score is given, which can be rated as “sufficient”(+), “insufficient” (–), or “indeterminate” (?). The rating depends on design, methods and outcomes of the validation study. When the necessary data to create a result was not reported or clearly specified, the “indeterminate” rating was given. An overview of the existing criteria can be found in Fig. 1.

### Grading the quality of evidence

In the third step of the analysis, the quality of evidence of all included studies were rated in order to assess how trustworthy the results are. For this step, the modified GRADE approach by the COSMIN group was used^5–7^.

The modified GRADE approach comprises four factors: (1) risk of bias (the methodological quality of the studies), (2) inconsistency (differences in results between studies that can’t be explained), (3) imprecision (total sample size of included studies), and (4) indirectness (evidence originates from other populations than the population of interest in the review).

Using the GRADE approach, evidence can be downgraded in case there are concerns regarding the quality (see Table 1a). Starting with the initial assumption of a high quality of the result, the quality of the evidence can be downgraded accordingly by up to three levels per factor if the criteria are not met.

**Table 1:** a: Definitions of the modified GRADE approach by COSMIN^5–7^; b: GRADE downgrading criteria for Risk of Bias^5–7^.

A Quality of evidence	Lower if
**High** = We are very confident that the true measurement property lies close to that of the estimate of the measurement property	***Risk of bias*** -1 Serious -2 Very serious -3 Extremely serious ***Inconsistency*** -1 Serious -2 Very serious ***Imprecision*** (*n* = sample size) -1 total *n* = 50–100 -2 total *n* < 50 ***Indirectness*** -1 Serious -2 Very serious
**Moderate** = We are moderately confident in the measurement property estimate: the true measurement property is likely to be close to the estimate of the measurement property, but there is a possibility that it is substantially different	
**Low** = Our confidence in the measurement property estimate is limited: the true measurement property may be substantially different from the estimate of the measurement property	
**Very low** = We have very little confidence in the measurement property estimate: the true measurement property is likely to be substantially different from the estimate of the measurement property	

B Risk of bias	Downgrading for Risk of Bias
**No**	There are multiple studies of at least adequate quality, or there is one study of very good quality available
**Serious**	There are multiple studies of doubtful quality available, or there is only one study of adequate quality
**Very serious**	There are multiple studies of inadequate quality, or there is only one study of doubtful quality available
**Extremely serious**	There is only one study of inadequate quality available

(1) Risk of Bias

To assess the quality of the evidence by using GRADE approach, we examined each RoB box according to the criteria specified in Table 1b. Following the “worst score counts” principle, the presence of a single box indicating an extremely serious risk of bias suffices for a maximum downgrade of three levels. Prerequisite for a downgrade is a definite result (i.e., a “+” or “−” rating, not a “?”) in the corresponding box in Step 2: Updated criteria of good measurement.

(2) Inconsistency

As we followed a qualitative approach, which did not include quantitative pooling (meta-analysis), we agreed on following criteria for downgrading: no downgrading the scale, if no or little inconsistency with valid explanation was found. One level (serious) downgrade, if little inconsistency without or moderate to high inconsistency with valid explanation was found. Two level (very serious) downgrade, if moderate to high inconsistency without explanation was found.

(3) Imprecision

Imprecision considers the total sample size across studies: a sample size of 50 < n < 100 results in a one level downgrade, while *n* < 50 results in a two-level downgrade.

(4) Indirectness

Indirectness may arise if the circumstances of an included study differ from what the review is aiming to examine, e.g. the study is done in another population than the population of interest or another context of use. In this review the population of interest are patients with schizophrenia or schizophrenia spectrum disorder. There was no downgrading, if patients of the comparison group had a different disease or were healthy.

#### Formulating recommendations and feasibility

Ultimately, the PROMs are classified into three categories based on the results of their evaluation:

(A) PROMs with evidence for sufficient content validity (any level) AND at least low quality evidence for sufficient internal consistency.

(B) PROMs categorized not in A or C.

(C) PROMs with high quality evidence for an insufficient measurement property.

In addition, the feasibility of the rating scale is examined in a descriptive way.

## Results

The search strategy resulted in 798 articles for QOLI, based on title and abstract we identified 28 articles and 4 articles were included in our review. For the LQOLP 488 articles were found, 36 articles were chosen based on title an abstract from which 8 were fit for our review. For the MANSA 353 articles were found, 19 articles were chosen by title and abstract and 4 articles were included. The re-performed literature search yielded 25 articles for QOLI, 18 articles for LQOLP and 32 articles for MANSA. None of the newly found articles could be included in our review. General characteristics of the included studies are presented in Table 2.

**Table 2:** Characteristics of included study population.

Study	Language	Country	Population	Mean (SD) Age	Gender (female in %)	Number of Patients
**Lehman’s Quality of Life Interview**						
Lehman (1983)	English	USA	Patients with mainly schizophrenia; also alcoholism, organic brain syndrome, affective disorder, substance abuse	42.1 (12.5)	34.5	278
Lehman (1988)	English	USA	Patients with mainly schizophrenia; also affective disorder, alcoholism, drug abuse, organic brain syndrom, mental retardation, personality disorder	LA: 42.1 (12.5) Rochester In: 38.5 (13.5) Rochester Out: 43.5 (15.0)	LA: 34.5 Rochester In: 47.5 Rochester Out: 57.1	LA: 278 Rochester In: 99 Rochester Out: 92
Lehman et al. (1993)	English	USA	Patients with schizophrenia, dementia, or additional criteria for persistent mental illness and disability met	38.1 (10.3)	46.6	59
Salyers et al. (2001)	English	USA	Patients with severe and persistent mental illness	39.4 (10.1)	43	202
**Lancashire Quality of Life Profile**						
Oliver et al. (1997)	English	UK, USA	NR	NR	NR	NR
Kaiser et al. (1997)	German, English	Germany, Wales	In- and outpatients with schizophrenia	Sample A: 30.2 (10.3) Samples B,E,F: 42.4–43.4 Sample C: 53.4 (14.0) Sample D: 49.0 (11.0)	Sample A: 71 Sample F: 32 Rest: 41–47	440
van Nieuwenhuizen et al. (1998)	Dutch	Netherland	In- and outpatients with schizophrenia spectrum disorder, bipolar disorder, major depression	36.9 (107)	40	40
Hansson et al. (1998)	Swedish	Sweden	Inpatients with psychosis	31.3 (range 18–69)	48.3	29
Gaite et al. (2000)	Dutch, Danish, English, Spanish, Italian	Netherland, Denmark, UK, Spain, Italy	In- and outpatients with schizophrenia	41.8 (11.1)	46	404
Ritsner et al. (2002)	Hebrew	Israel	Inpatients with schizophrenia, schizoaffective disorder, mood disorder	38.9 (10.1)	25.1	199
Zahid et al. (2009)	Arab	Kuwait	Outpatients with schizophrenia	44.1 (13.1)	31.5	130
Su et al. (2017)	Chinese	Taiwan	Inpatients with schizophrenia	49.2 (7.8)	34	100
**Manchester Short Assessment of Quality of Life**						
Priebe et al. (1999)	English	UK	Patients with schizophrenia, bipolar-affective psychosis, depression, obsessive compulsive disorder, anxiety disorder	40.9 (14.9)	NR	55
Björkman et al. (2005)	Swedish	Sweden	Patients with schizophrenia, other psychosis, non psychosis	47 (range 29–68)	53	92
Kusel et al. (2007)	English	UK	In- and outpatients with schizophrenia spectrum disorder	NR	NR	442
Lange et al. (2022)	Dutch	Netherlands	Patients with schizophrenia, schizoaffective disorder	67.9 (7.4)	64	107

Below we describe the COSMIN evaluation of each scale, starting with the QOLI^2^, followed by LQOLP^3^ and MANSA^4^.

In Table 3 an overview of all risk of bias and updated criteria ratings are presented.

**Table 3:** Overview of the results of QOLI, LQOLP and MANSA.

Measurement property (No. of studies assessing measurement property)	COSMIN Risk of Bias				Updated criteria of good measurement		
	Very good	Adequate	Doubtful	Inadequate	+	−	?
**Lehman’s Quality of Life Interview**							
PROM development (*n* = 1)	0	0	0	1			
Content validity (*n* = 0)							
Structural validity (*n* = 1)	0	0	0	1	0	0	1
Internal consistency (*n* = 3)	3	0	0	0	0	0	3
Reliability (*n* = 3)	0	0	1	2	0	1	2
Hypotheses testing for construct validity *- convergent validity* (*n* = 1)	0	1	0	0	0	1	0
**Lancashire Quality of Life Profile**							
PROM development (*n* = 1)	0	0	0	1			
Content validity (*n* = 0)							
Structural validity (*n* = 1)	0	0	0	1	0	0	1
Internal consistency (*n* = 8)	5	0	0	3	0	0	8
Reliability (*n* = 4)	0	2	2	0	0	2	2
Hypotheses testing for construct validity *- convergent validity* (*n* = 2)	0	2	0	0	1	2	0
Hypotheses testing for construct validity *- known-groups validity* (*n* = 1)	0	0	1	0	1	0	0
**Manchester Short Assessment of Quality of Life**							
PROM development (*n* = 0)							
Content validity (*n* = 0)							
Structural validity (*n* = 1)	0	1	0	0	0	0	1
Internal consistency (*n* = 2)	2	0	0	0	0	0	2
Criterion validity (*n* = 1)	1	0	0	0	1	0	0
Hypotheses testing for construct validity *- convergent validity* (*n* = 3)	0	3	0	0	1	2	0

### Lehman’s quality of life interview^2^

#### Assessing the risk of bias

##### Content validity

Content validity provides information on whether a PROM actually measures the construct it intends to and whether the content is relevant, comprehensive and understandable for the target population. It includes the PROM development and the actual content validity.

##### PROM development

Information on PROM development was found for QOLI^2^. It received an “inadequate” rating as no formal qualitative approach was used to analyse data on concept elicitation and comprehensibility in the pilot study.

##### Content validity

In a content validity study patients and professionals are asked about relevance, comprehensiveness and comprehensibility of the final form of the PROM. No study on content validity could be included in our review.

##### Structural validity

Structural validity evaluates how well the scores of a scale represent the underlying construct it is intended to measure. This property is applicable only to scales that are based on a reflective model, where all items or subscales are presumed to be indicators of a single latent construct and are therefore expected to correlate. As all three instruments aim to measure an underlying latent construct (quality of life), they can be considered reflective models, making structural validity a relevant property for evaluation.

The structural validity study for QOLI^12^ showed “inadequate” reporting quality as no appropriate statistical method was used, e.g., confirmatory (CFA) or exploratory factor analysis (EFA).

##### Internal consistency

All three studies^2,12,13^ reporting on internal consistency of QOLI received a “very good” rating for calculating Cronbach’s alpha (CA) for each subscale.

##### Reliability

Reliability measures if the score of an instrument is consistent under specific circumstances, while the study population is stable. This could be test-retest reliability or interrater reliability.

For the QOLI three studies reported on test-retest reliability^2,13,14^. Two studies^13,14^ with “inadequate” and one^2^ with a “doubtful” rating due to inappropriate time intervals or unclear invariance of the test conditions.

##### Hypotheses testing for construct validity

The construct validity assesses if the scores of a PROM meet with hypotheses that were installed regarding other instruments or differences between relevant groups, provided that it accurately measures the intended construct. The Updated COSMIN Risk of Bias Checklist^5–7^ defines two parts for the “Hypotheses Testing for Construct Validity” section. The first one is convergent validity, which evaluates the comparisons of the original PROM with other outcome measurements. The second one is known-groups validity, which assesses comparisons between relevant groups.

##### Convergent validity

It must be noted that no universal definition of QOL exists that has been agreed on. Constructs with varying degrees of relatedness are expected to show differing correlation. Similar constructs include (sub-)scales of instruments measuring Health-related QOL. Conceptually related constructs included measurements on depression. Dissimilar, but related constructs are instruments on mania or positive and negative symptoms (for further details see updated criteria).

One paper^14^ presented data on convergent validity for QOLI, receiving an “adequate” rating.

#### Updated criteria for good measurement properties

##### Structural validity

The structural validity of QOLI was reported by one study^12^ using factor analysis and difference-score reliabilities. The result received an indeterminate “?” rating, because no detailed data was reported and information on results of CTT, Rasch or IRT were missing.

##### Internal consistency

The result of all three studies^2,12,13^ reporting on internal consistency of QOLI received an indeterminate “?” rating as the criteria for “at least low evidence for sufficient structural validity” were not met.

##### Reliability

The results of two^2,14^ of the three studies assessing test-retest reliability of the QOLI were rated indeterminate “?”. Instead of calculating intraclass correlation coefficient (ICC) or weighted Kappa, as proposed by COSMIN, they used Pearson correlation. The results of the third study^13^ received an insufficient “−” rating, because ICC was < 0.7 for two of the eight satisfaction scales.

##### Hypotheses testing for construct validity

Following the COSMIN Risk of Bias manual four hypotheses were formulated for assessing convergent validity. To receive a sufficient rating the following hypotheses must be met.

1. Correlations with instruments measuring similar or conceptually related constructs should be ≥ 0.50.
2. Correlations with instruments measuring related, but dissimilar constructs should be lower, that is, 0.30 to 0.50.
3. Correlations with instruments measuring unrelated constructs should be < 0.30.
4. Correlations defined under 1, 2, and 3 should differ by a minimum of 0.10.

For the evaluation of convergent validity, we included two types of comparisons: total score comparisons between the included scales and reference scales, and subscale comparisons between analogous subscales of instruments measuring similar constructs. Comparisons between subscales and total scores of different instruments, or between subscales measuring unrelated constructs, were excluded, as no meaningful hypotheses about the expected direction and magnitude of such correlations could be formulated. Following COSMIN, for a sufficient “+” rating, 75% of the hypotheses had to apply.

The one study for QOLI^14^ compared corresponding subscales of Heinrich-Carpenters Quality of Life scale and QOLI. Overall, the results received an insufficient “−” rating.

#### Modified GRADE approach

1. For structural validity there was extremely RoB, as only one study of inadequate quality was included. This alone leads to a maximum −3 downgrade, which suggests the quality of evidence is very low. For internal consistency no downgrade was given as 3 studies of very good quality were included. Only 2 studies with inadequate and one with doubtful quality were included for reliability, meaning there is very serious risk of bias with a −2 downgrade which leads to low quality of evidence. For convergent validity the inclusion of only one study with adequate quality leads to a −1 downgrade for serious RoB and moderate quality of evidence.
2. No downgrade for any measurement property was given for inconsistency.
3. For imprecision, only the total sample size for convergent validity was too low, therefore an additional −1 downgrade leaded to a low quality of evidence for convergent validity.
4. With most of the study population across the measurement properties classified as severely mentally ill, no downgrade was given for indirectness.

Apart from internal consistency, the GRADE approach indicated low to very low quality of evidence, meaning that the true values of the measurement properties for QOLI may differ substantially from those obtained in this review.

### Lancashire quality of life profile^3^

#### Assessing the risk of bias

##### PROM development

One study^15^ reported on PROM development for LQOLP. It received an “inadequate” rating as it failed to assess comprehensiblitiy and comprehensiveness of the PROM in the pilot studies.

##### Structural validity

One study^16^ on structural validity for LQOLP was found. It received an “inadequate” rating, as the minimum requirement for sample size to perform factor analysis was not attained in the study.

##### Internal consistency

For LQOLP 8 studies^3,16–22^ reported on internal consistency. 5^18–22^ receiving a “very good” rating, 3^3,16,17^ a “inadequate” rating as internal consistency was calculated using an inadequate approach^3^ or not calculated for each subscale separately^16,17^.

##### Reliability

Four studies^19–22^ reported test-retest reliability for LQOLP. Two^20,21^ with “adequate” and two^19,22^ with “doubtful” rating, because it was unclear whether patients were stable between tests and reliability was measured using Pearson or Spearman correlation instead of preferred ICC.

##### Convergent validity

Two studies^21,22^ were reported for LQOLP, both with “adequate” ratings for reporting quality.

##### Known-groups validity

One study^21^ reported on known-groups validity for LQOLP. It was examined whether LQOLP could reliably discriminate between patients and non-patients. It received a “doubtful” rating as the comparison group also differed in gender and relationship status.

#### Updated criteria for good measurement properties

##### Structural validity

One study^16^ reported on structural validity for the LQOLP using EFA. The results were rated indeterminate “?” due to no existing criteria for EFA by COSMIN.

##### Internal consistency

The results of all 8 studies^3,16–22^ reporting on internal consistency of LQOLP received an indeterminate “?” rating as the criteria for “at least low evidence for sufficient structural validity” were not met.

##### Reliability

The results of two^19,22^ of the four studies on test-retest reliability of LQOLP were deemed indeterminate “?” as either of them used Pearson correlation as a measure. The results of the other two studies^20,21^ obtained an insufficient “−” rating as many of the subscales did not reach the demanded ICC ≥ 0.7.

##### Convergent validity

Two studies for LQOLP^21,22^ reported for total score comparisons. The results for one^21^ were sufficient “+” and for the other one^22^ insufficient “−”. One study^21^ evaluated comparisons of analogous subscales of the Quality of Life Enjoyment and Satisfaction Questionnaire and the results were rated insufficient “−”.

##### Known-groups validity

The objective of known-groups validity is to examine whether comparisons between known subgroups adhere to predefined hypotheses.

The results of the one study^21^ reporting on known-groups validity for the LQOLP received a sufficient “+” rating.

#### Modified GRADE approach

1. For structural validity there was extremely RoB, as only one study of inadequate quality was included. This alone leads to a maximum -3 downgrade, which suggests the quality of evidence is very low. No downgrade was given for internal consistency as several studies with very good quality were reported. For reliability there are multiple studies of doubtful quality, which leads to serious RoB and a downgrade of -1. With 2 studies of adequate quality no downgrade was given for convergent validity, whereas for known-groups validity a very serious RoB resulted in a -2 downgrade.
2. No downgrade for any measurement property was given for inconsistency.
3. No downgrade for imprecision was given for any measurement property.
4. With the majority of the study population classified as schizophrenia, no downgrade was given for indirectness.

Only for internal consistency and reliability high and moderate quality of evidence was found. Otherwise, the GRADE approach indicated low to very low quality of evidence, meaning that the true values of the measurement properties for LQOLP may differ substantially from those obtained in this review.

### Manchester short assessment of quality of life^4^

#### Assessing the risk of bias

##### Structural validity

One study^23^ reported on structural validity of MANSA. The reporting quality was rated “adequate”, because EFA as only second-best statistical method was used and the ideal sample size included in the study could not be reached.

##### Internal consistency

Two studies for MANSA^4,24^ on internal consistency received a “very good” rating.

##### Criterion validity

Criterion validity assesses if a PROM is an adequate reflection of its gold standard. According to COSMIN a gold standard can be the long version of a shortened PROM.

In our review MANSA is a short version that has been derived from its long version, the LQOLP. One study^4^ evaluated the criterion validity of MANSA, rated with “very good”.

##### Convergent validity

Three studies^4,24,25^ on convergent validity were included for MANSA, all of them receiving an “adequate” rating.

#### Updated criteria for good measurement properties

##### Structural validity

One study^23^ reported on structural validity for the LQOLP using EFA, which identified a three-factor model. The results were rated indeterminate “?” due to no existing criteria for EFA by COSMIN.

##### Internal consistency

The results of the two studies^4,24^ reporting on internal consistency of MANSA received an indeterminate “?” rating as the criteria for “at least low evidence for sufficient structural validity” were not met.

##### Criterion validity

All of the correlations between satisfaction ratings between MANSA and LQOLP exceeded 0.7 for the study on criterion validity^4^. Hence, the results are sufficient “+”.

##### Convergent validity

All three studies^4,24,25^ for MANSA were examining total score comparisons. The results of two^24,25^ of them were insufficient “−” and one^4^ sufficient “+”.

#### Modified GRADE approach

1. For structural validity there was serious RoB as only one study of adequate quality was included, resulting in a −1 downgrade and moderate quality of evidence. No downgrade was given for internal consistency, criterion validity and convergent validity.
2. Inconsistency in the results of convergent validity was found resulting in a −1 downgrade.
3. A downgrade of −1 for criterion validity was given as the total sample size for this measurement property was rather low.
4. With the majority of the study population classified as schizophrenic, no downgrade was given for indirectness.

Only for internal consistency high quality of evidence was found. For criterion and convergent validity the quality of evidence was moderate meaning that we are confident in the obtained results of the measurement property in this review, but there is a possibility that they could differ substantially.

## Discussion

This systematic review provides a comprehensive evaluation of three widely used instruments designed to assess QOL in patients with schizophrenia: QOLI, LQOLP, and MANSA. To our knowledge, this is the first time the updated COSMIN guidelines have been applied for a review of these instruments. In total, 17 relevant validation studies were included in the review.

PROMs in category A generate reliable results and can therefore be recommended for use. PROMs in category B cannot be definitively recommended or advised against, further research is necessary for recommendation. PROMs in category C are not recommended for use.

None of the three PROMs included in this review could be assigned to category A. This is primarily attributable to the absence of content validity studies for any of the included PROMs, which represents a prerequisite for this categorization. Furthermore, the second prerequisite - at least low-quality evidence for sufficient internal consistency - was equally not fulfilled. Internal consistency ratings across all PROMs were indeterminate, as the necessary condition for a determinate rating, namely at least low-quality evidence for structural validity, had not been established.

Regarding the QOLI, five validation studies were included in this review. Ultimately, this PROM was assigned to category B, as the criteria for neither category A nor category C were fulfilled. Classification into category C would have required high-quality evidence for an insufficient measurement property; however, as the majority of results across measurement properties were either indeterminate or the quality of evidence was downgraded, this threshold was not reached. The indeterminate ratings and lower quality of evidence can be partly attributed to the use of outdated statistical methods that do not meet the requirements of the COSMIN standards, a relatively recent and stringent methodology. Furthermore, multiple validation studies^26,27^ had to be excluded from this review, as they employed modified versions of the QOLI, partly without providing adequate explanation of the modifications applied.

In terms of the LQOLP, eight studies were identified as eligible for inclusion in this review. Based on the COSMIN evaluation, the LQOLP was assigned to category B, as the criteria for neither category A nor category C were met. The available studies yielded predominantly indeterminate results or low-quality evidence across measurement properties. As high-quality evidence for an insufficient measurement property could not be established, classification into category C was not warranted.

For MANSA only four validation studies could be included in this review. MANSA was assigned to category B, as no high quality evidence for an insufficient measurement property was found, which would have lead to category C. With four studies addressing only 4 of the 10 measurement properties, the overall evidence base for this PROM remains notably sparse.

There are also aspects of feasibility of the reviewed instruments that need to be addressed. The high number of items in the QOLI and LQOLP (but not MANSA) leads to long administration times, which negatively affects their feasibility. This extended duration can be particularly challenging for individuals with schizophrenia, as maintaining concentration and motivation over such a lengthy period may be difficult. As a result, data quality may suffer due to respondent fatigue, reduced engagement, or incomplete responses. These limitations restrict the practicality of both instruments in time-limited clinical environments and research settings.

Our review has limitations. Due to the heterogeneity and sparsity of the included studies, quantitative pooling was not feasible. This limits the precision of our estimates but reflects the current state of the evidence base rather than a methodological shortcoming of this review.

Up to this date, a consensus on a standardised definition of QOL and its dimensions remains elusive. Previous work done by van Krugten^8^ and Connel^9,10^ helped us choose instruments that included domains rated relevant for people with mental health problems. Nevertheless, it should be noted that the PROMs included in this review could only partially cover the relevant domains evaluated by these reports.

Our group has only focused on domain- and disease-specific QOL instruments so far^11^ (further publications in progress). This decision was made because specific aspects of quality of life may be important for people with schizophrenia^28^. However, since generic QOL instruments are broadly used in schizophrenia research, it would be interesting to see how good these instruments perform in a schizophrenia sample.

In conclusion, the continued use of the instruments evaluated in this review should be considered with caution. Based on the currently available evidence, their routine application cannot be confidently recommended. There seem to exist more promising instruments, such as the Schizophrenia Quality of Life Scale^29^ and its revision, the Schizophrenia Quality of Life Scale Revision 4^30^. Despite the fact that these instruments did also not perform flawlessly according to COSMIN standards and received a (B) rating in a related review^11^, they have been assessed with more up-to-date methods and hold higher potential to be recommended if further studies are conducted on their limitations.

## Data Availability

We do not have individual patient data. All ratings can be found in the tables.

## References

[1] Gladis, M. M. et al. Quality of life: expanding the scope of clinical significance. *J. Consult Clin. Psychol.* **67**, 320–31 (1999). 10369052 10.1037//0022-006x.67.3.320

[2] Lehman, A. F. A quality of life interview for the chronically mentally ill. *Eval. Program Plan.* **11**, 51–62 (1988).

[3] Oliver, J. P. et al. Measuring the quality of life of severely mentally ill people using the Lancashire Quality of Life Profile. *Soc. Psychiatry Psychiatr. Epidemiol.* **32**, 76–83 (1997). 9050348 10.1007/BF00788924

[4] Priebe, S. et al. Application and results of the Manchester Short Assessment of Quality of Life (MANSA). *Int J. Soc. Psychiatry* **45**, 7–12 (1999). 10443245 10.1177/002076409904500102

[5] Mokkink, L. B. et al. COSMIN Risk of Bias checklist for systematic reviews of patient-reported outcome measures. *Qual. Life Res.* **27**, 1171–1179 (2018). 29260445 10.1007/s11136-017-1765-4 PMC5891552

[6] Prinsen, C. A. C. et al. COSMIN guideline for systematic reviews of patient-reported outcome measures. *Qual. Life Res.* **27**, 1147–1157 (2018). 29435801 10.1007/s11136-018-1798-3 PMC5891568

[7] Terwee, C. B. et al. COSMIN methodology for evaluating the content validity of patient-reported outcome measures: a Delphi study. *Qual. Life Res.* **27**, 1159–1170 (2018). 29550964 10.1007/s11136-018-1829-0 PMC5891557

[8] van Krugten, F. C. W. et al. Instruments to assess quality of life in people with mental health problems: a systematic review and dimension analysis of generic, domain- and disease-specific instruments. *Health Qual. Life Outcomes* **19**, 249 (2021). 34727928 10.1186/s12955-021-01883-w PMC8561965

[9] Connell, J. et al. Quality of life of people with mental health problems: a synthesis of qualitative research. *Health Qual. Life Outcomes* **10**, 138 (2012). 23173689 10.1186/1477-7525-10-138 PMC3563466

[10] Connell, J., O’Cathain, A. & Brazier, J. Measuring quality of life in mental health: are we asking the right questions?. *Soc. Sci. Med.* **120**, 12–20 (2014). 25194472 10.1016/j.socscimed.2014.08.026 PMC4224500

[11] Zúñiga Le-Bert, M. et al. Schizophrenia Quality of Life Scale and Schizophrenia Quality of Life Scale Revision 4: a Systematic Review of Measurement Properties. *Schizophr. Bull.* **51**, 997–1008 (2025). 39036973 10.1093/schbul/sbae119 PMC12236313

[12] Lehman, A. F. The effects of psychiatric symptoms on quality of life assessments among the chronic mentally ill. *Eval. Program Plann* **6**, 143–51 (1983). 10265063 10.1016/0149-7189(83)90028-9

[13] Salyers, M. P. et al. Reliability of instruments in a cooperative, multisite study: employment intervention demonstration program. *Ment. Health Serv. Res.* **3**, 129–39 (2001). 11718205 10.1023/a:1011519514465

[14] Lehman, A. F., Postrado, L. T. & Rachuba, L. T. Convergent validation of quality of life assessments for persons with severe mental illnesses. *Qual. Life Res* **2**, 327–33 (1993). 8136797 10.1007/BF00449427

[15] Bridges, K., Huxley, D. P., Huxley, P., Mohamad, H., & Oliver, J., *Quality of Life and Mental Health Services**(**1st ed*.*)*. 1996: Routledge.

[16] Zahid, M. A. et al. Correlates of quality of life in an Arab schizophrenia sample. *Soc. Psychiatry Psychiatr. Epidemiol.* **45**, 875–87 (2010). 19727531 10.1007/s00127-009-0131-4

[17] Kaiser, W. et al. Profiles of subjective quality of life in schizophrenic in- and out-patient samples. *Psychiatry Res.* **66**, 153–66 (1997). 9075279 10.1016/s0165-1781(96)02958-7

[18] van Nieuwenhuizen, C. et al. The Lancashire quality of life profile: first experiences in The Netherlands. *Community Ment. Health J.* **34**, 513–24 (1998). 9793741 10.1023/a:1018794530481

[19] Hansson, L., Svensson, B. & Björkman, T. Quality of life of the mentally ill. Reliability of the Swedish version of the Lancashire quality of life profile. *Eur. Psychiatry* **13**, 231–4 (1998). 19698631 10.1016/S0924-9338(98)80010-2

[20] Gaite, L. et al. Quality of life in schizophrenia: development, reliability and internal consistency of the Lancashire quality of life profile—European Version. EPSILON Study 8. European Psychiatric Services: inputs linked to outcome domains and needs. *Br. J. Psychiatry* ***177****,* s49–54 (2000). 10.1192/bjp.177.39.s49 10945078

[21] Ritsner, M. et al. Subjective quality of life in severely mentally ill patients: a comparison of two instruments. *Qual. Life Res.* **11**, 553–61 (2002). 12206576 10.1023/a:1016323009671

[22] Su, C. T., Yang, A. L. & Lin, C. Y. Comparing two schizophrenia-specific quality of life instruments in institutionalized people with schizophrenia. *Psychiatry Res.* **258**, 274–282 (2017). 28860017 10.1016/j.psychres.2017.08.053

[23] Lange, S. M. M. et al. The 5-year outcome of subjective quality of life in older schizophrenia patients. *Qual. Life Res* **31**, 2471–2479 (2022). 35067820 10.1007/s11136-021-03062-2

[24] Björkman, T. & Svensson, B. Quality of life in people with severe mental illness. Reliability and validity of the Manchester Short Assessment of quality of life (MANSA). *Nord J. Psychiatry* **59**, 302–6 (2005). 16195135 10.1080/08039480500213733

[25] Kusel, Y. et al. Measurement of quality of life in schizophrenia: a comparison of two scales. *Soc. Psychiatry Psychiatr. Epidemiol.* **42**, 819–23 (2007). 17762904 10.1007/s00127-007-0249-1

[26] Russo, J. et al. Longitudinal assessment of quality of life in acute psychiatric inpatients: reliability and validity. *J. Nerv. Ment. Dis.* **185**, 166–75 (1997). 9091598 10.1097/00005053-199703000-00006

[27] Barry, M. M. & Crosby, C. Quality of life as an evaluative measure in assessing the impact of community care on people with long-term psychiatric disorders. *Br. J. Psychiatry* **168**, 210–6 (1996). 8837912 10.1192/bjp.168.2.210

[28] Papaioannou, D., Brazier, J. & Parry, G. How valid and responsive are generic health status measures, such as EQ-5D and SF-36, in schizophrenia? A systematic review. *Value Health* **14**, 907–20 (2011). 21914513 10.1016/j.jval.2011.04.006 PMC3179985

[29] Wilkinson, G. et al. Self-report quality of life measure for people with schizophrenia: the SQLS. *Br. J. Psychiatry* **177**, 42–6 (2000). 10945087 10.1192/bjp.177.1.42

[30] Martin, C. R. & Allan, R. Factor structure of the Schizophrenia Quality of Life Scale Revision 4 (SQLS-R4). *Psychol. Health Med.* **12**, 126–34 (2007). 17365893 10.1080/13548500500407383

